# Our State Associations

**Published:** 1874-07

**Authors:** 


### Editorial.

OUR STATE MEDICAL ASSOCIATIONS hold their regular annual convocations that their members may mingle freely with each other in professional and social intercourse; that they may soften down somewhat the asperities which constitute the natural growth of isolation; that they may exchange freely thought, opinion and experience touching the manifold questions which arise in the daily practice of their vocation; that they may put upon record whatever is new and valuable in the general summing up of the previous year's professional labor.

The artist, in picturing upon canvas some colossal monument, finds himself utterly unable to fasten upon his work the idea of grandeur which fills his own imagination until he falls upon the happy expedient of presenting a man, or some familiar animal, at its base, that the eye of the observer may be able to draw the contrast. Great and small are purely relative terms.

We must be able to institute a comparison in the mind, or we wholly fail to appreciate size. Nothing can be truly great unless there be something smaller; nothing can be small except as we imbibe with it the idea of something larger, with which we are enabled to institute a comparison. The mass of medical practitioners in this country follow their vocation in isolated localities, where opportunities for professional intercourse are limited. Each, in his sphere, is seldom brought in contact with a mind which may presume to arrogate to itself equality with his own in matters pertaining to his calling. In things medical he is the authority, he is the court of last appeal, he is the monarch of absolute sway, whose fiat mortal man disobeys at the peril of his life. He is a giant when compared with the intellectual pygmies which surround him, and there can be no limit affixed to his greatness because there is nothing greater by which to measure him.

In any department of life man can only progress as he is stimulated to do so by contact with his fellow-man. Isolate him so that he is deprived of all means of measuring himself with others of his species, and the incentive to progress is lost, he ceases at once to grow. Bring him out into the world, place him in the company of tall men and he instinctively begins to tip-toe and stretch up a little. By nature man is prone to think well of himself, to place a liberal estimate upon his own powers, his own greatness, and to discount somewhat the pretensions of others. He likes to perambulate with small men that he may look down complacently and patronisingly upon them, satisfied with his own stature and feeling need of nothing more. Such is human nature—so are we constituted.

The value of the Medical Association as a leveling institution, in which the small men are taught to recognize their true status and stimulated to growth upon the one hand, whilst the tall men find themselves side by side with others of equal stature, and learn to abate somewhat their arrogance upon the other hand, should not be too lightly estimated. It is difficult in the one individual to compare the strength of one power or faculty of the mind with another of the same mind; but when the comparison is instituted with other individuals we discover a soft spot here, a weak spot there, and are thereby stimulated the better to fortify ourselves. Since no two persons can occupy the same point of space at any one time, there must always be a greater or less angle formed by the lines of vision of any two observers of the same object. So, too, in mental phenomena, the inherent differences in the minds of different individuals must ever give rise to variations in the impressions derived from the same exterior influence, or the conclusions drawn from the same premises. The observation or conclusion of one mind is absolute and irrefutable until called in question by the differing observation or conclusion of another, and it is only by the comparison of numerous and repeated observations that the truth is reached.

The field of medical observation and research has become so vast in modern times that no one mind, however strong and comprehensive, is able to compass it all. There must always be something which the strongest man lacks, and which the weakest man may possess. Bring the two together; each may learn from the other; the strong man discovers his weak point and essays to fortify it, the weak man takes courage and is strengthened by the contact. The eminent French inventor of the chain ecraseur and the gum-elastic drainage tube, Chassaignac, once went into ecstacies over the ingenuity which flattened a squirrel-shot and punctured it with an awl, to fasten the metallic suture in its place.

Industrious and progressive men in our ranks generally appreciate the advantage of association in stimulating medical progress, and look forward with pleasure to the annual convocation as a season of recreation, of enjoyment and of profit which they would not willingly forego. But so abject is the slavery of our profession to the public necessity and the public caprice it is difficult to lay aside a busy practice even for the brief period required for attendance upon the Association. Prof. Fleetwood Churchill, of Dublin, once expressed his astonishment at the number of medical men leaving active practice at home to spend many months in Europe for medical observation and study, and remarked: "I have longed for years to spend two or three weeks upon the continent, to look through the French and German hospitals, but I dare not do it. It would require years to repair the damage to my business." Although we are not in this country so absolute slaves to our patrons, still we all feel the obstacle to the indulgence of our inclinations in this direction. Very many must depend for their enjoyment and profit of medical associations upon the printed transactions of those bodies, which they are enabled to read at home in the intervals of business.

It has been customary with medical, as well as other societies of learned men, to publish from time to time each its volume of transactions, in order that absent members, and others interested, may have access to the scientific truths which they contain, and maintain their interest in the bodies which issue them, as well as to put upon permanent record the results of their brain labor. In former times these transactions were preserved in manuscript among the archives, and a weary pilgrimage to some scientific Mecca was required if one would avail himself of the precious lore therein enrolled. These records were eagerly sought by inquiring minds, and lost little of their interest by lapse of time. But the world turned around, the printers' art superseded the classical manuscripts in velum, the vehicle took the place of the sandal, and, by long and tedious stages, the coveted volume reached the foot-sore pilgrim at his own fireside along with his annual gazette of current intelligence. Again the world turned over; with improvement in the construction of the hull and trim of the sails monthly news crossed the ocean. Another revolution and the weekly newspaper became a public necessity; then came steam and the daily press, until at last the evening edition must furnish food for the mind already tired of that supplied at noon, and science has in our day harnessed the lightning itself to the car of human progress.

However much we may value the traditions of our art, however much we may pride ourselves upon the conservatism of our cloth, it behooves us, if we would maintain our Medical Associations, to keep up somewhat with the progressive spirit of the age in which we live. Truly are the children of the world wiser in their day and generation than the children of light. There is much which we might profitably learn from those intent upon the ignoble pursuit of sordid gold as the great end of life, if we would but open our eyes and look around us.

There is perhaps no one respect in which our medical bodies exhibit their persistent Rip Van Winkle-ism than in their sleepy volumes of dead transactions, which are yearly laid away to accumulate dust and harbor the moth. The annual session of the body gathers together a moiety of its members who enjoy the freshness of the occasion, and by way of penalty therefor pay into the treasury a yearly sum that the cold meats and stale bread may be hashed up, with long months of accumulated mould, to be spurned at last by those who remained at home.

If our firm grasp upon the traditions of the past cannot be relaxed at once, if we must for years to come submit to the annual infliction of the musty volume, it would seem that a wise economy would dictate that it should be cheaply made from a simple block of substantial *beech-wood,* only lettered and ornamented to the accustomed standard.

It is found to be impracticable to levy and collect a yearly tax upon the members of our associations at large; the funds of the body must be raised almost wholly from those who are already taxed with the expense of attendance upon its meetings. This annual assessment would supply the individual assessed with one or more of the standard medical periodicals of our day, and lay upon his table promptly the journal of proceedings of the State Association, to be quickly followed by all that is of value in the way of contributed papers, and this withont expense to the body itself. Absent members would by this means, too, be supplied with the information they desire as quickly as the press can furnish it, and their interest in the Association itself enlivened and permanently maintained. As regards the preservation of the published papers, and their readiness of access in future years, all that is desirable in these respects is more likely of attainment in the columns of a medical journal, making up a good volume for annual binding, than in the paper-covered pamphlet usually issued. If we would infuse life and interest into our Associations let the dummy volume in cheap and lasting wood receive the dust of the top shelf, while we give promptly to the medical world the living transactions through the vigilant and active periodical press.

